# Mathematical Ecology Analysis of Geographical Distribution of Soybean-Nodulating Bradyrhizobia in Japan

**DOI:** 10.1264/jsme2.ME13079

**Published:** 2013-11-16

**Authors:** Yuichi Saeki, Sokichi Shiro, Toshiyuki Tajima, Akihiro Yamamoto, Reiko Sameshima-Saito, Takashi Sato, Takeo Yamakawa

**Affiliations:** 1 Department of Biochemistry and Applied Biosciences, Faculty of Agriculture, University of Miyazaki, Miyazaki 889–2192, Japan; 2 Interdisciplinary Graduate School of Agriculture and Engineering, University of Miyazaki, Miyazaki 889–2192, Japan; 3 Faculty of Agriculture, Shizuoka University, Shizuoka 422–8529, Japan; 4 Faculty of Bioresource Sciences, Akita Prefectural University, Akita 010–0195, Japan; 5 Faculty of Agriculture, Kyushu University, Fukuoka 812–8581, Japan

**Keywords:** *Bradyrhizobium*, community structure, niche, latitude, *Rj*-genotype soybean

## Abstract

We characterized the relationship between the genetic diversity of indigenous soybean-nodulating bradyrhizobia from weakly acidic soils in Japan and their geographical distribution in an ecological study of indigenous soybean rhizobia. We isolated bradyrhizobia from three kinds of *Rj*-genotype soybeans. Their genetic diversity and community structure were analyzed by PCR-RFLP analysis of the 16S–23S rRNA gene internal transcribed spacer (ITS) region with 11 *Bradyrhizobium* USDA strains as references. We used data from the present study and previous studies to carry out mathematical ecological analyses, multidimensional scaling analysis with the Bray-Curtis index, polar ordination analysis, and multiple regression analyses to characterize the relationship between soybean-nodulating bradyrhizobial community structures and their geographical distribution. The mathematical ecological approaches used in this study demonstrated the presence of ecological niches and suggested the geographical distribution of soybean-nodulating bradyrhizobia to be a function of latitude and the related climate, with clusters in the order Bj123, Bj110, Bj6, and Be76 from north to south in Japan.

Soybeans (*Glycine max.* L. Merr.) establish a symbiotic relationship with soybean-nodulating bacteria during the development of their nitrogen-fixing organs, the root nodules. The major soybean-nodulating rhizobia that have been identified are *Bradyrhizobium japonicum*, *Bradyrhizobium elkanii*, and *Ensifer/Sinorhizobium fredii* (4, 13, 14, 18, 37, 47). Additional species of soybean-nodulating rhizobia have also been discussed extensively in the literature because of the complexity of their taxonomic classification (10, 23, 41, 43, 45). Major symbionts of soybeans for nodulation in acidic-neutral soil in Japan are *B. japonicum* and *B. elkanii* (21, 28, 36, 40). Soybean-nodulating bacteria are found throughout wide regions of the world, and their genetic diversity may reflect geographical and climatic differences as well as the diversity of local hosts. Therefore, analysis of the genetic diversity and field distributions of indigenous soybean-nodulating rhizobia is important for improving our understanding of rhizobial ecology under various conditions.

Our previous research (28, 31) demonstrated the genetic diversity and field distributions of indigenous soybean-nodulating bradyrhizobia isolated from weakly acidic soils from five fields in Japan and suggested that indigenous bradyrhizobia cluster in association with strains listed in north-to-south order as follows: *B. japonicum* USDA 123, 110, 6^T^, and *B. elkanii* USDA 76^T^. Suzuki *et al.* (40) reported the dominance of *B. elkanii* strains in weakly acidic-neutral soils of the islands of Okinawa, Miyakojima and Ishigaki and the dominance of *S. fredii* strains in alkaline soils of the Okinawa island. Risal *et al.* (25) reported the genetic diversity of native soybean bradyrhizobia isolated from different topographical regions along the southern slopes of the Himalayan Mountains in Nepal. Furthermore, Adhikari *et al.* (1) demonstrated the genetic diversity and distribution of soybean-nodulating bradyrhizobia in relation to climate and soil properties in Nepal. Li *et al.* (19) reported that the genetic diversity and biogeography of soybean rhizobia were related to soil determinant factors such as pH, electrical conductivity (EC), and phosphorus content in Hebei Province, China. These results suggest that a relationship exists between the genetic diversity and field distribution of indigenous soybean-nodulating rhizobia and soil properties such as temperature (as influenced by latitude and altitude), phosphorus content, EC, and soil pH.

In the host soybean, the genes related to nodulation, the *Rj* genes, are known as nodulation regulatory genes, and *Rj* genotypes of *rj*_1_, *Rj*_2_, *Rj*_3_, *Rj*_4_ and non-*Rj*, which lack these genetical phenotypes, have been confirmed to exist naturally (6). Soybean cultivars harboring *Rj* genes are involved in the inhibition of effective nodulation by certain serogroups of rhizobia as well as in the preferential selection of appropriate rhizobia for nodulation (11, 20, 26). Thus, in an analysis of indigenous soybean-nodulating bacteria, it is necessary to use several types of *Rj* genotypes of soybean cultivars for the isolation of rhizobia.

To examine the relationship between bradyrhizobial genetic diversity and their geographical distribution in an ecological study of indigenous soybean rhizobia, we used three types of *Rj*-genotype soybeans to isolate indigenous soybean-nodulating bradyrhizobia from weakly acidic soils in Japan. We investigated their genetic diversity, community structure, and allocation among fields by PCR-RFLP analysis of internal transcribed spacer (ITS) regions of the 16S–23S rRNA gene. Furthermore, we used data from the present and previous studies to conduct mathematical ecological analyses to clarify the relationship between the community structures of soybean-nodulating bradyrhizobial and their geographical distributions.

## Materials and Methods

### Soil samples

Soil samples for the isolation of soybean-nodulating bradyrhizobia were collected from experimental fields of domestic research institutes, Akita Prefectural University (Ogata, Akita, Japan), Yamanashi Prefectural Agritechnology Center (Kai, Yamanashi, Japan), Shizuoka University (Fukuroi and Fujieda, Shizuoka, Japan), Kochi University (Nangoku, Kochi, Japan), Kyushu University (Kasuya, Fukuoka, Japan), and Kagoshima Prefectural Institute for Agricultural Development, Tokunoshima branch (Tokunoshima, Kagoshima, Japan). These soils have a history of soybean cultivation without any bradyrhizobial inoculation. Table 1 provides information about the soil samples collected during this study and previous studies (28, 40).

### Isolation of soybean-nodulating rhizobia

For isolation of indigenous soybean-nodulating bacteria, the soybean cultivars Akishirome or Bragg (non-*Rj*), CNS (*Rj*_2_*Rj*_3_), and Fukuyutaka (*Rj*_4_) were grown in experimental soil placed in vermiculite culture pots amended with a nitrogen-free nutrient solution (26) at 40% (v/v) water content in a growth chamber (day: 28°C for 16 h, night: 23°C for 8 h) for 4 weeks. After cultivation, the roots were washed thoroughly with tap water. The nodules were collected randomly and surface-sterilized with 70% ethanol and a dilute sodium hypochlorite solution (0.25% available chlorine) followed by rinsing with sterile distilled water. Each nodule homogenate was streaked onto a yeast-extract mannitol agar (YMA) (42) plate and incubated for 5–7 days in the dark at 28°C. To determine the genus of the isolates, a single colony was streaked onto YMA plates containing 0.002% (w/v) bromothymol blue (15) and incubated as described above. After incubation, each isolate was maintained on YMA medium at 4°C. To eliminate the possibility of contamination with irrelevant soybean-nodulating bacteria, we confirmed that there was no nodule formation on the negative control soybean plants without soil. Sixty isolates, 20 isolates for each *Rj*-genotype soybean, were considered to be a soybean-nodulating rhizobial community for each experimental soil. A total of 780 isolates from 13 sites, except for sample B sites (Akita B, Shizuoka B, and Okinawa B), were used in multivariate analyses. Datasets from the sample B sites were used to verify the results of multiple regression analysis. Compatibility with host soybean of representative isolates from soil samples in each cluster after cluster analysis was confirmed by an inoculation test as described previously (28).

### PCR-RFLP analysis of the 16S-23S rRNA gene ITS region

Each isolate was cultured in 1.0 mL Hepes-Mes medium (5) supplemented with 0.1% L-arabinose (33) for 3–4 days at 28°C. Total DNA for the PCR template was extracted from the cell pellet with bacteria lysate buffer according to the method described by Hiraishi *et al.* (9). PCR-RFLP analysis of the ITS region was carried out using *Ex Taq* DNA polymerase (Takara Bio, Otsu, Japan) and the ITS primer set (BraITS-F: 5′-GACTGGGGTGAAGTCGTAAC-3′, BraITS-R: 5′-ACGTCCTTCATCGCCTC-3′), and four restriction enzymes, *Hae*III, *Hha*I, *Msp*I, and *Xsp*I (Takara Bio), as described previously (28). The restricted fragments were separated and screened with QIAxcel capillary electrophoresis apparatus (Qiagen, Hilden, Germany). As reference strains, we used *B. japonicum* USDA 4, 6^T^, 38, 110, 115, 123, 124, and 135, and *B. elkanii* USDA 46, 76^T^, and 94 (27).

### Cluster analysis

The sizes of the fragments on electrophoresis were measured with a 50-bp ladder marker (Nippon Gene, Toyama, Japan) and the fragment sizes calculated from the sequence data of the reference strains. All reproducible fragments longer than 50 bp were used for cluster analysis, and some irreproducible fragments were excluded. The genetic distance between pairs of isolates (*D*) was calculated using the following equation:

(1) $$D_{\text{AB}}=1-[2N_{\text{AB}}/(N_{\text{A}}+N_{\text{B}})]$$

where *N*_AB_ represents the number of RFLP bands shared by strains A and B, and *N*_A_ and *N*_B_ represent the numbers of RFLP bands in the two strains (22, 32). The *D* values for all pairs of isolates were calculated. Cluster analysis was carried out using the unweighted-pair group method using arithmetic averages. A dendrogram was constructed with PHYLIP software program ver. 3.6 (J. Felsenstein, University of Washington).

### Multidimensional scaling analysis and polar ordination analysis

To characterize relationships among the bradyrhizobial communities, we performed multidimensional scaling (MDS) analysis based on the Bray-Curtis index as a measure of dissimilarity (3). The Bray-Curtis index has been characterized as one of the indices that best reflects properties between communities (7). We calculated the Bray-Curtis index (*BC*) with the following equation:

(2) $$BC_{\text{AB}}=[\Sigma |n_{\text{A}}-n_{\text{B}}|]/[\Sigma \;(n_{\text{A}}+n_{\text{B}})]$$

where *BC*_AB_ is the dissimilarity between communities A and B, and *n*_A_ and *n*_B_ represent the number of isolates belonging to clusters associated only with communities A and B, respectively. Three-dimensional (3-D) MDS analyses based on the Bray-Curtis index were conducted using the command “cmdscale” in R software program ver. 2.12.1 (24).

To determine the relative distances among the bradyrhizobial communities based on 3-D MDS plots of the communities in the 3-D Euclidean space, we used the Euclidean distances between communities based on a trigonometric figure to conduct polar ordination and plotted the distances between the polar axes (17, 29, 44). The bradyrhizobial communities isolated from the Ishigaki Island site (24.38°N) and the Hokkaido site (42.89°N) were regarded as polar samples (*i.e.*, 100% difference) because the difference in their latitudes and distance between soil sample sites was the greatest. The Euclidean distances (*Ed*), each pole point and other points on the 3-D MDS plots were calculated with the x-, y-, and z-axis coordinates of the points with the following equation:

(3) $$Ed_{\text{AB}}={\left[{\left(X_{\text{A}}-X_{\text{B}}\right)}^{2}+{\left(Y_{\text{A}}-Y_{\text{B}}\right)}^{2}+{\left(Z_{\text{A}}-Z_{\text{B}}\right)}^{2}\right]}^{1/2}$$

where *Ed*_AB_ is the linear distance between communities A and B on the 3-D MDS plots, and *X*_A_ and *X*_B_, *Y*_A_ and *Y*_B_, and *Z*_A_ and *Z*_B_ represent the coordinates on the x-, y-, and z-axes of communities A and B, respectively. The distances from the each pole were converted to percentage differences (%), *D*_1_ and *D*_2_, from the polar communities Ishigaki and Hokkaido. Simultaneous equations were constructed from the trigonometric figure using the Pythagorean theorem as described previously (29). Parameter *x* represents the polar difference (%) from the 0% pole (Ishigaki site) and is calculated as follows (44):

(4) $$x=(L^{2}+{D_{1}}^{2}-{D_{2}}^{2})/2L$$

where *D*_1_ and *D*_2_ are the percentage differences between a particular bradyrhizobial community and the communities at the Ishigaki site and Hokkaido site, respectively. Parameter *L* is the difference between the two polar sites (i.e. 100%). The relationships between the polar ordination and the latitudes of the field sampling sites were estimated. For estimation of community structure distribution, this analysis was conducted with the combined data from the experimental soil sites as well as with each datum of *Rj*-genotype soybeans from the experimental soil sites.

### Multiple regression analysis

To characterize the coordinates from the regression equation in terms of the coordinates obtained from latitude and polar ordination analysis, we used major clusters in the soybean-nodulating bacterial communities as explanatory independent variables to conduct multiple regression analysis (MRA). Clusters Bj6, Bj110, Bj123, and Be76, which accounted for more than 10% of the total number of isolates (Table 2), were used as the major clusters for independent variables in the MRA. For characterization of the relationships between the coordinates obtained from polar ordination analysis and the latitude of sample soil sites, MRA was conducted with four major cluster data: nodule occupancy (%) of Bj6, Bj110, Bj123, and Be76, using the command “lm” in R software program ver. 2.12.1. These cluster data were used as independent variables against the coordinate calculated from the regression equation as the dependent variable. The equation for the MRA results was verified with sample B sites: Akita B, Shizuoka B, and Okinawa B. Further analyses of MDS and polar ordination were conducted for each *Rj*-genotype soybean data set.

## Results

### PCR-RFLP analysis of the 16S-23S rRNA gene ITS region

**Fig. 1:**
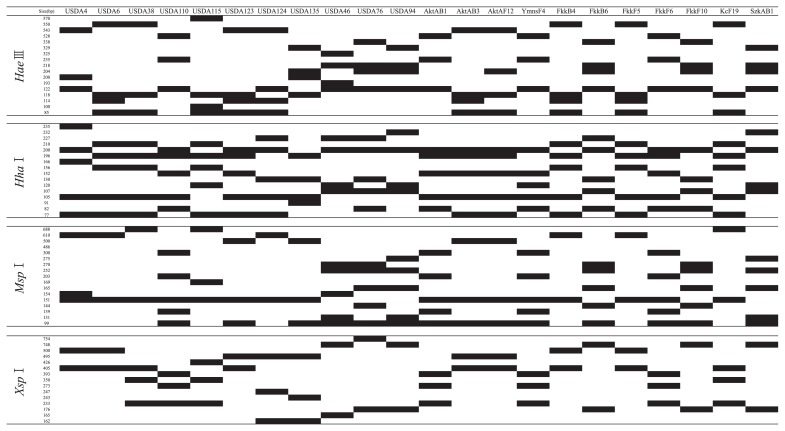
Schematic representation of electrophoresis patterns of PCR-RFLP of 16S–23S rRNA gene ITS region of soybean-nodulating bradyrhizobia isolated in this study.

**Fig. 2:**
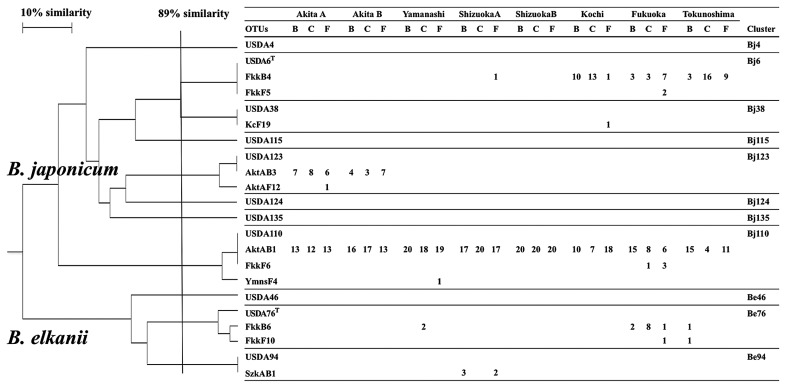
Dendrogram of PCR-RFLP analysis of 16S–23S rRNA gene ITS region of soybean-nodulating bradyrhizobia. B, C, F indicate host soybean cultivars, Bragg or Akishirome (non-*Rj*), CNS (*Rj*_2_*Rj*_3_) and Fukuyutaka (*Rj*_4_), respectively.

Fig. 1 is a schematic representation of the electrophoresis fragment patterns from RFLP analysis. Fifteen RFLP patterns associated with operational taxonomic units (OTUs) were detected from indigenous bradyrhizobia isolated from Akita A, Akita B, Yamanashi, Shizuoka A, Shizuoka B, Fukuoka, Kochi, Tokunoshima, and reference strains. Cluster analysis was conducted for the 15 OTUs containing the 11 RFLP patterns of the reference strains. Fig. 2 shows the dendrogram of the indigenous bradyrhizobia. A similarity of 89% was the maximum similarity used to distinguish between USDA 6^T^ and USDA 38, the strain with the most closely related RFLP patterns among the reference strains. This similarity was applied as the criterion for cluster analysis, and 11 clusters were detected in the dendrogram. Six of the 11 clusters were generated from indigenous isolates. However, isolates belonging to 5 clusters (Bj4, Bj115, Bj124, Bj135, and Be46) were not detected among the indigenous isolates in this study. Eight clusters (Bj) belonged to the cluster of *B. japonicum*, and 3 clusters (Be) belonged to those of *B. elkanii*. Four of the 8 clusters, Bj6, Bj38, Bj110, and Bj123, showed RFLP patterns that were identical or similar to those of *B. japonicum* strains USDA 6^T^, 38, 110, and 123, respectively. Clusters Be76 and Be94 showed RFLP patterns identical or similar to those of *B. elkanii* strains USDA 76^T^ and USDA 94, respectively. The clusters of *B. elkanii* evinced relatively lower variance than those of *B. japonicum*. Table 2 lists the isolates belonging to clusters detected in this study and previous studies.

### Multidimensional scaling and polar ordination analysis

**Fig. 3:**
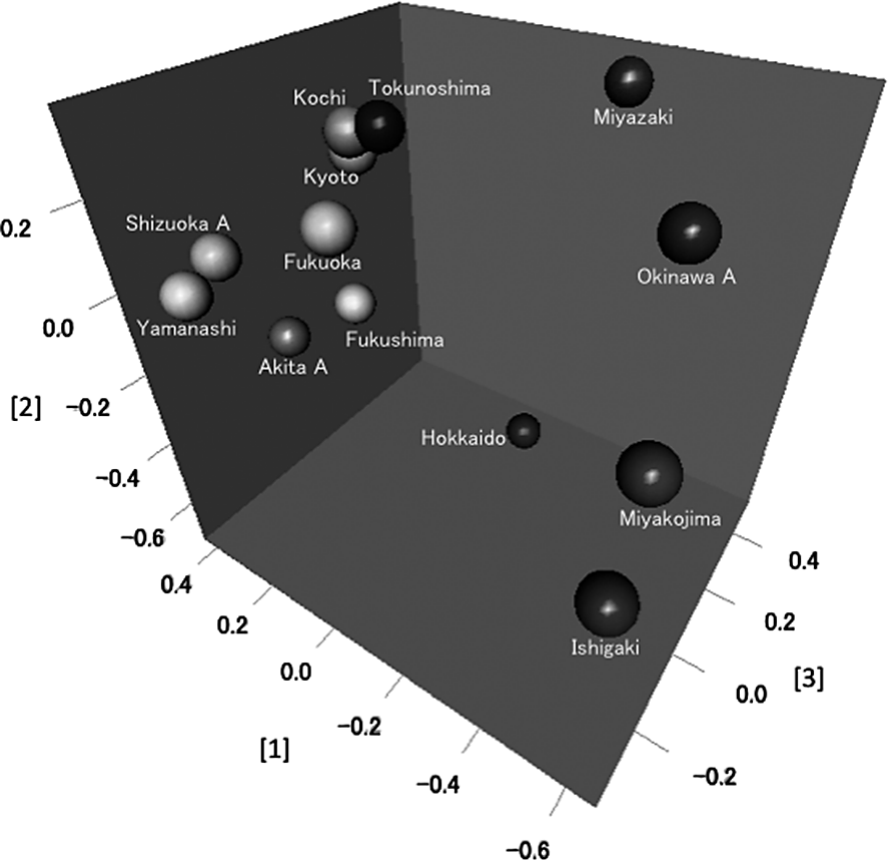
Plots of soybean-nodulating bradyrhizobial communities by 3-dimensional multidimensional scaling analysis based on Bray-Curtis index. The coordinates of axis [1], [2], [3] are as follows: Hokkaido: −0.0536, −0.6712, 0.3637, AkitaA: 0.3808, −0.2725, −0.0389, Fukushima: 0.2706, −0.2026, 0.0841, Yamanashi: 0.3544, −0.0153, −0.2845, Kyoto: 0.2039, 0.1995, 0.0669, ShizuokaA: 0.3567, 0.0303, −0.1975, Fukuoka: 0.0594, 0.4652, −0.1613, Kochi: 0.1905, 0.2465, 0.0480, Miyazaki: −0.2057, 0.3042, 0.4660, Tokunoshima: 0.1310, 0.2618, 0.0712, OkinawaA: −0.5003, 0.2144, 0.0825, Miyakojima: −0.5901, −0.0402, −0.1922, Ishigaki: −0.5975, −0.2203, −0.3079, respectively.

**Fig. 4:**
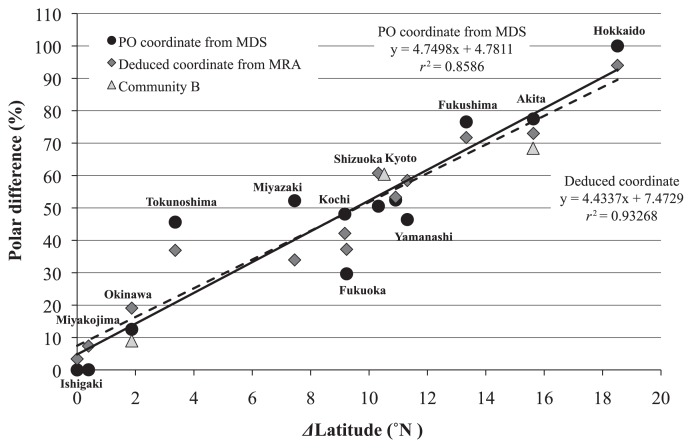
Relationship between soybean-nodulating bradyrhizobial community and latitude of the soil sampling site. Two linear regression equations are shown in the figure. The equation of the polar ordination coordinate from MDS indicates the regression equation from the result of polar ordination analysis of MDS plots, and the equation of the deduced coordinate indicates the regression equation from coordinates of multiple regression equation, Y = 0.213Bj123 + 0.130Bj110 − 0.592Bj6 − 0.712Bj76 + 73.42 (*R*^2^ = 0.9014, F4,8 = 28.42, *p* <0.0001), for the equation from the polar ordination coordinate from MDS as independent variables.

Fig. 3 shows the results of 3-D MDS and the coordinates of the axes obtained from the 3-D MDS analysis. Table 3 shows the results of polar ordination and indicates the percentage differences of each soil site from the Ishigaki site (0%) to the Hokkaido site (100%). The relationships between the polar ordination of bacterial communities and the northern latitudes of the sample field sites are indicated by the filled circles in Fig. 4. There was a high degree of correlation (*r*^2^ = 0.8586) between the change in latitude and the polar ordination coordinate from MDS.

### Multiple regression analysis

There were four major clusters of soybean-nodulating bradyrhizobia: Bj6, Bj110, Bj123, and Be76 (Table 2). These four clusters were therefore used as independent variables to explain the dependent variables in the MRA. Equation (5) was constructed from the coefficients obtained from the MRA, the adjusted coefficient of determination being 0.9014 (Fig. 4).

(5) Y=0.213·Bj123+0.130·Bj110-0.592·Bj6-0.712·Be76+73.42

where Bj123, Bj110, Bj6, and Be76 are the percentages of occupancy of the clusters in the soybean nodules. Data from the sample B sites (Akita B, Shizuoka B, and Okinawa B) were used for verification of the equation that resulted from the MRA. The coordinates of the B sample sites, indicated by the triangles in Fig. 4, were calculated using the equation about as well as the coordinates of the sample A sites.

### Multidimensional scaling and polar ordination analysis for each Rj-genotype

**Fig. 5:**
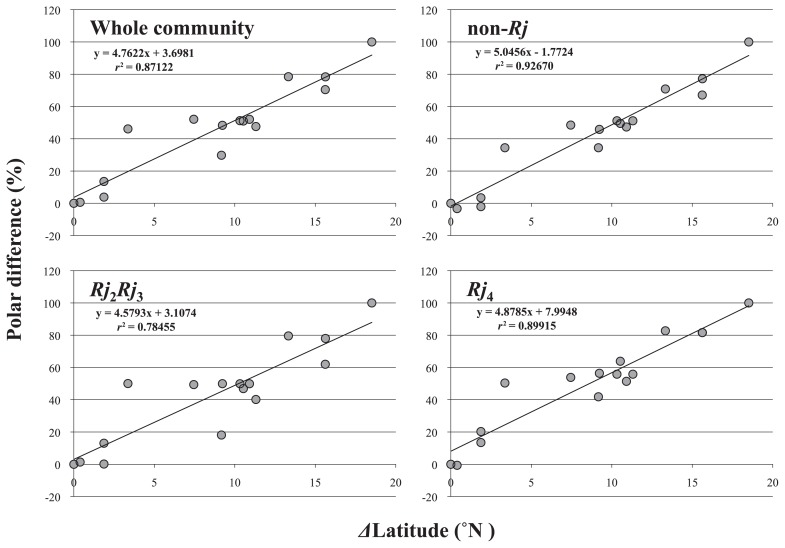
Relationship between soybean-nodulating bradyrhizobial community of each *Rj*-genotype soybean and latitude of the soil sampling site.

Fig. 5 shows the relationships between the polar ordination analysis of the coordinates of the 3-D MDS analysis from each *Rj*-genotype soybean and the northern latitudes of the sample field sites. These figures were constructed from all data including community B, for each *Rj*-genotype soybean. Analytical procedures were identical to those described above. Fig. 5 evinces a high degree of correlation between changes in latitude and polar differences of soybean-nodulating bradyrhizobial communities in the case of non-*Rj* genotype (*r*^2^ = 0.927) and the *Rj*_4_-genotype (*r*^2^ = 0.899), whereas the corresponding correlation in the case of the *Rj*_2_*Rj*_3_-genotype was relatively low (*r*^2^ = 0.785). The *r*^2^ of the same relationship for the whole community containing these three genotypes was 0.871.

## Discussion

The results of this study suggested that major soybean-nodulating bradyrhizobia in Japan are isolates that belong to clusters of Bj6, Bj110, and Bj123, and Be76 (Tables 2, 3, and Fig. 4). Although our previous study (28) suggested that the major soybean-nodulating rhizobial clusters in Japan included clusters of Bj6, Bj38, Bj110, Bj123, and Be76, the results of this study indicate that the Bj38 cluster may be excluded from the major clusters of soybean-nodulating rhizobia in Japan. Although the variances of nodule occupancy rates of clusters were large, it is likely that the nodule occupancy rates of these four clusters vary with the northern latitude (Table 3 and Fig. 4).

In general, soybean cultivation seasons depend on the region and cultivar. Experimental results have demonstrated that the structure of soybean-nodulating rhizobial communities will depend on cultivation temperatures even on identical soil samples (20, 38). The expression level of the *nod* genes of bradyrhizobia is also dependent on temperature and differs among strains (46). Furthermore, it has been suggested that population occupancy rates of bradyrhizobia in soil are affected by soil temperature. For example, *B. elkanii* USDA 76^T^ becomes dominant under high temperature conditions, and *B. japonicum* USDA 123 becomes dominant under low temperature conditions (30). These results suggest that soybean-nodulating bradyrhizobia may be characterized by temperature-dependent ecological niches for infection of the host root and population occupancy in the soil. As a result, bradyrhizobia might evince changes of population occupancy and community structures in soil as habitat changes with latitude.

In previous studies, *B. elkanii* isolates were detected with high frequencies from southern regions of Japan (28, 40). However, isolation rates of *B. elkanii* from Gray Lowland soils and Gley soils were relatively low, and dominant isolates belonged to the Bj110 cluster in this study (Table 2). A high frequency of Bj110 was also detected from fine-particle Red Yellow soils on the island of Tokunoshima, which is located in southern Japan. In the United States, indigenous soybean-bradyrhizobia belonging to Bj123 cluster are dominant in northern regions, and Be clusters are dominant in central and southern regions (16, 39). The Bj110 cluster is detected in central regions, but in association with few bradyrhizobia. These results suggest that soil chemical and/or physical properties determined by soil texture (e.g., silt versus clay) might affect the indigenization and/or nodulation of soybean-nodulating bradyrhizobia. Anaerobic conditions developed under paddy field conditions in fine-particle soils such as Gray Lowland soils might be suitable for indigenization of strains such as USDA110 in temperate climate regions. This reason for the high occupancy of the Bj110 cluster in fine-particle soils might be strain capability for denitrification. Bradyrhizobia show denitrification activity. Although the end products of denitrification depend on the strain capability, *B. japonicum* strain USDA110 possesses a full set of functional denitrifying genes and reduces NO_3_^−^ to N_2_ (35). This function of the strain might work advantageously to become dominant in the soils. This strain also shows denitrifying capability to reduce N_2_O surrounding the soybean root system (34). Recently, Itakura *et al.* (12) demonstrated the mitigation of N_2_O emission from soils by inoculation of soils with *B. japonicum* USDA110 under field conditions. Therefore, utilization of useful bradyrhizobia that show high N-fixing and full denitrifying capabilities is important not only for increasing yields but also for environmental conservation in agriculture.

Additionally, as environmental factors for bradyrhizobial ecology, it is reported that soil chemical properties such as pH, phosphorus and climate conditions affect the indigenization of the genera and species of soybean rhizobia (1, 19, 25). Our previous study (40) also indicated that among the genera of soybean-nodulating rhizobia, the presence of the genera *Bradyrhizobium* and *Sinorhizobium* can depend on the soil pH. It has also been reported that soybean cultivation management practices affect the genetic variance of soybean-nodulating bacteria (2, 8). Furthermore, differences in the diversity of communities of soybean-nodulating bradyrhizobia related to *Rj* genotypes of the host soybean cultivars were demonstrated (20, 29, 38). Although difference among community structures depending on *Rj* genotypes may depend on the community structure of soybean-nodulating rhizobia in the soil, the relatively low coefficient of determination for the *Rj*_2_*Rj*_3_ genotype in polar ordination analysis based on MDS plots (Fig. 5) supports the previous results (20, 29). These results suggest the possibility of formulating a model of the community structure of soybean-nodulating rhizobia based on latitude, soil texture, soil chemical/physical properties such as temperature, pH, EC, oxidation-reduction potential, soil management, and soybean *Rj* genotypes.

In this study, we investigated the rhizobial community structure via the analysis of soybean-nodulating bradyrhizobia, and demonstrated an ecological niche along latitude in Japan. To elucidate bradyrhizobial ecology, it will be necessary to analyse model experiments using microcosms containing representative strains, considering soil types and texture, soil chemical/physical properties, soil management practices, and soybean cultivars. Furthermore, we must develop more direct methods to characterize rhizobial communities in soil, *i.e.* methods using soil DNA, to advance our understanding of indigenous rhizobial ecology and for further construction of reliable models of soybean-nodulating rhizobial community structure.

## Figures and Tables

**Table 1:** List of soil samples used in this study

Denotation of sample soil	Soil group^a^	Sampling site	Latitude, Longitude	*Δ* latitude (°N)	pH (H_2_O)	EC (dSm^−1^)	Reference
Hokkaido	Andosols	Memuro, Hokkaido, Japan	42.89N, 143.07E	18.51	5.2	0.17	Saeki *et al.* 2006
Akita A	Gley soils	Ogata, Akita, Japan	40.01N, 139.98E	15.63	6.1	0.06	This study
Akita B	Gley soils	Ogata, Akita, Japan	40.00N, 139.96E	15.62	5.9	0.05	This study
Fukushima	Andosols	Arai, Fukushima, Japan	37.71N, 140.39E	13.33	5.0	0.05	Saeki *et al.* 2006
Yamanashi	Gray Lowland soils	Kai, Yamanashi, Japan	35.68N, 138.49E	11.31	6.1	0.07	This study
Kyoto	Andosols	Ayabe, Kyoto, Japan	35.29N, 135.26E	10.91	5.1	0.15	Saeki *et al.* 2006
Shizuoka A	Gray Lowland soils	Fukuroi, Shizuoka, Japan	34.70N, 137.93E	10.32	5.3	0.18	This study
Shizuoka B	Gley soils	Fujieda, Shizuoka, Japan	34.90N, 138.27E	10.52	5.8	0.46	This study
Fukuoka	Gray Lowland soils	Kasuya, Fukuoka, Japan	33.61N, 130.46E	9.23	5.6	0.02	This study
Kochi	Gray Lowland soils	Nangoku, Kochi, Japan	33.55N, 133.68E	9.17	4.9	0.34	This study
Miyazaki	Andosols	Kibanadai, Miyazaki, Japan	31.83N, 131.42E	7.45	5.7	0.06	Saeki *et al.* 2006
Tokunoshima	Red Yellow soils	Tokunoshima, Kagoshima, Japan	27.74N, 128.97E	3.36	7.3	0.06	This study
Okinawa A	Red Yellow soils	Nishihara, Okinawa, Japan	26.25N, 127.76E	1.87	4.7	0.06	Suzuki *et al.* 2008
Okinawa B	Red Yellow soils	Nishihara, Okinawa, Japan	26.25N, 127.76E	1.87	5.7	0.04	Suzuki *et al.* 2008
Miyakojima	Dark Red soils	Miyakojima, Okinawa, Japan	24.77N, 125.33E	0.39	7.5	0.05	Suzuki *et al.* 2008
Ishigaki	Red Yellow soils	Ishigaki, Okinawa, Japan	24.38N, 124.19E	0.00	6.1	0.03	Suzuki *et al.* 2008

a Classification of Cultivated Soils in Japan (Third Approximation).

**Table 2:** Cluster and number of soybean-nodulating bradyrhizobia from each soil sample and *Rj*-genotype soybeans

Cluster	Bj6	Bj38	Bj115	Bj110	Bj123	BjH	BjF	BjO	Be61	Be76	Be94	BeO	Reference

Sample													
Hokkaido					58 (19, 19, 20)	2 (1, 1, 0)							Saeki *et al.* 2006
Akita A				38 (13, 12, 13)	22 (7, 8, 7)								This study
Akita B				46 (16, 17, 13)	14 (4, 3, 7)								This study
Fukushima		20 (7, 1, 12)	3 (1, 0, 2)	22 (10, 10, 2)	12 (2, 8, 2)		2 (0, 1, 1)			1 (0, 0, 1)			Saeki *et al.* 2006
Yamanashi				58 (20, 18, 20)						2 (0, 2, 0)			This study
Kyoto	14 (8, 3, 3)	8 (5, 0, 3)	6 (2, 1, 3)	29 (5, 13, 11)							3 (0, 3, 0)		Saeki *et al.* 2006
Shizuoka A	1 (0, 0, 1)			54 (17, 20, 17)							5 (3, 0, 2)		This study
Shizuoka B				60 (20, 20, 20)									This study
Fukuoka	15 (3, 3, 9)			33 (15, 9, 9)						12 (2, 8, 2)			This study
Kochi	24 (10, 13, 1)	1 (0, 0, 1)		35 (10, 7, 18)									This study
Miyazaki	40 (16, 7, 17)	7 (4, 0, 3)									13 (0, 13, 0)		Saeki *et al.* 2006
Tokunoshima	28 (3, 16, 9)			30 (15, 4, 11)						2 (2, 0, 0)			This study
Okinawa A	25 (7, 6, 12)							1 (0, 1, 0)		25 (11, 8, 6)	1 (1, 0, 0)	8 (1, 5, 2)	Suzuki *et al.* 2008
Okinawa B	16 (4, 2, 10)									41 (15, 18, 8)	2 (0, 0, 2)	1 (1, 0, 0)	Suzuki *et al.* 2008
Miyakojima	8 (3, 3, 2)									49 (16, 16, 17)	1 (0, 1, 0)	2 (1, 0, 1)	Suzuki *et al.* 2008
Ishigaki									1 (0, 0, 1)	59 (20, 20, 19)			Suzuki *et al.* 2008
No. isolate	171	36	9	405	106	2	2	1	1	191	25	11	960
Percentage (%)	17.8	3.8	0.9	42.2	11.0	0.2	0.2	0.1	0.1	19.9	2.6	1.1	100

Upper number in each column indicates whole number of isolates and lower indicates number of isolates from non-*Rj*, *Rj*_2_*Rj*_3_, and *Rj*_4_, respectively.

**Table 3:** Data for multiple regression analysis and coordinates calculated

Site	Dependent variable		Independent variable (Nodule occupancy %)				*Δ* latitude (°N)	Coordinate of MRA equation^c^
	
	PO coordinate from MDS^a^	Coordinate of regression equation^b^	Bj123	Bj110	Bj6	Be76		
Hokkaido	100.0	92.6	96.7	0.0	0.0	0.0	18.51	94.0
Akita A	77.5	78.9	36.7	63.3	0.0	0.0	15.63	73.0
Fukushima	76.6	68.0	20.0	36.7	0.0	1.7	13.33	71.7
Yamanashi	46.4	58.4	0.0	96.7	0.0	3.3	11.31	58.5
Kyoto	52.4	56.5	0.0	48.3	23.3	0.0	10.91	53.3
Shizuoka A	50.5	53.8	0.0	90.0	1.7	0.0	10.32	60.7
Fukuoka	29.7	48.5	0.0	55.0	25.0	20.0	9.23	37.2
Kochi	48.1	48.3	0.0	58.3	40.0	0.0	9.17	42.2
Miyazaki	52.2	40.1	0.0	0.0	66.7	0.0	7.45	34.0
Tokunoshima	45.6	20.7	0.0	50.0	46.7	3.3	3.36	36.9
Okinawa A	12.5	13.6	0.0	0.0	41.7	41.7	1.87	19.1
Miyakojima	0.1	6.6	0.0	0.0	13.3	81.7	0.39	7.4
Ishigaki	0.0	4.8	0.0	0.0	0.0	98.3	0.00	3.4

Akita B			23.3	76.7	0.0	0.0	15.62	68.4
Shizuoka B			0.0	100.0	0.0	0.0	10.52	60.4
Okinawa B			0.0	0.0	26.7	68.3	1.87	9.0

a Coordinates calculated by Polar Ordination based on coordinates of MDS analysis.

b Coordinates calculated by the linear regression equation; y = 4.7498x + 4.7811, as shown in Fig. 4.

c Coordinates calculated by multiple regression equation; Y = 0.213Bj123+0.130Bj110−0.592Bj6−0.712Bj76+73.42 as shown in equation (5).
